# Mdm2 RING Mutation Enhances p53 Transcriptional Activity and p53-p300 Interaction

**DOI:** 10.1371/journal.pone.0038212

**Published:** 2012-05-29

**Authors:** Hilary V. Clegg, Yoko Itahana, Koji Itahana, Sundhar Ramalingam, Yanping Zhang

**Affiliations:** 1 Lineberger Comprehensive Cancer Center, School of Medicine, University of North Carolina at Chapel Hill, Chapel Hill, North Carolina, United States of America; 2 Department of Radiation Oncology, School of Medicine, University of North Carolina at Chapel Hill, Chapel Hill, North Carolina, United States of America; 3 Department of Pharmacology, School of Medicine, University of North Carolina at Chapel Hill, Chapel Hill, North Carolina, United States of America; 4 Curriculum in Genetics and Molecular Biology, School of Medicine, University of North Carolina at Chapel Hill, Chapel Hill, North Carolina, United States of America; 5 Cancer and Stem Cell Biology Program, Duke-NUS Graduate Medical School Singapore, Singapore, Singapore; German Cancer Research Center, Germany

## Abstract

The p53 transcription factor and tumor suppressor is regulated primarily by the E3 ubiquitin ligase Mdm2, which ubiquitinates p53 to target it for proteasomal degradation. Aside from its ubiquitin ligase function, Mdm2 has been believed to be capable of suppressing p53's transcriptional activity by binding with and masking the transactivation domain of p53. The ability of Mdm2 to restrain p53 activity by binding alone, without ubiquitination, was challenged by a 2007 study using a knockin mouse harboring a single cysteine-to-alanine point mutation (C462A) in Mdm2's RING domain. Mouse embryonic fibroblasts with this mutation, which abrogates Mdm2's E3 ubiquitin ligase activity without affecting its ability to bind with p53, were unable to suppress p53 activity. In this study, we utilized the *Mdm2^C462A^* mouse model to characterize in further detail the role of Mdm2's RING domain in the control of p53. Here, we show *in vivo* that the Mdm2^C462A^ protein not only fails to suppress p53, but compared to the complete absence of Mdm2, Mdm2^C462A^ actually enhances p53 transcriptional activity toward p53 target genes *p21/CDKN1A*, *MDM2*, *BAX*, *NOXA*, and *14-3-3σ*. In addition, we found that Mdm2^C462A^ facilitates the interaction between p53 and the acetyltransferase CBP/p300, and it fails to heterodimerize with its homolog and sister regulator of p53, Mdmx, suggesting that a fully intact RING domain is required for Mdm2's inhibition of the p300-p53 interaction and for its interaction with Mdmx. These findings help us to better understand the complex regulation of the Mdm2-p53 pathway and have important implications for chemotherapeutic agents targeting Mdm2, as they suggest that inhibition of Mdm2's E3 ubiquitin ligase activity may be sufficient for increasing p53 activity *in vivo*, without the need to block Mdm2-p53 binding.

## Introduction

The p53 tumor suppressor protein is frequently mutated in cancer, with approximately 50% of cancers containing mutations that inactivate p53 itself, and many of the remaining cancers thought to harbor mutations that otherwise inactivate the p53 tumor suppressor pathway [1]. In response to DNA damage and other stimuli, p53 induces cell cycle arrest or apoptosis by transcriptionally activating genes that control these processes. In healthy cells, it is essential that p53's activity be kept in check so that the normal cell cycle can proceed. This control of p53 is accomplished primarily by the E3 ubiquitin ligase Mdm2 (murine double minute 2) [2].

Mdm2 has long been thought to inactivate p53 in two ways: by ubiquitinating p53 to induce its degradation, and by binding with p53 to conceal its transactivation domain. Mdm2 serves as an E3 ubiquitin ligase that conjugates a chain of ubiquitin molecules onto p53, targeting p53 for proteasome-mediated degradation [3], [4], [5]. In addition, Mdm2 binds with a region of p53 that overlaps with its transactivation domain, and many *in vitro* and/or overexpression studies supported the idea that Mdm2 binding alone, without ubiquitination, could suppress p53's transactivational activity [6], [7], [8].

A recent study by Itahana et al. [9] using an Mdm2 knockin mouse challenged the notion that Mdm2 is capable of suppressing p53 activity through binding alone. In that study, a knockin mouse was generated in which a single cysteine-to-alanine point mutation (C462A) was introduced into Mdm2's RING domain in order to abrogate Mdm2's E3 ubiquitin ligase activity without affecting the protein's ability to bind with p53 [10], [11]. Using this mouse model, designated as *Mdm2^C462A/C462A^* (hereafter referred to as *Mdm2^m/m^*), the separate contributions of Mdm2's E3 ubiquitin ligase activity and its ability to bind with p53 could be analyzed *in vivo* under conditions of endogenous protein expression. Using mouse embryonic fibroblast (MEF) cells obtained from this model, Itahana et al. showed that the Mdm2^C462A^ protein was capable of binding with p53 yet could not ubiquitinate p53 nor elicit its degradation [9].

While this work suggested that Mdm2-p53 binding alone, without ubiquitination, is not capable of inhibiting p53's activity, two issues became apparent: first, the expression of only one p53 target, aside from Mdm2 itself, was examined, and second, it was not shown that the mutant Mdm2 retained the ability to interact with p53 while on a target gene promoter. The study here aimed to address these concerns and further characterize the contribution of Mdm2's RING domain in suppressing p53. We show that Mdm2^C462A^ indeed interacts with p53 on the p21 promoter and that Mdm2^C462A^ fails to suppress transcription of multiple p53 targets, including p21, Mdm2, Bax, Noxa, and 14-3-3σ. Interestingly, we found that Mdm2-p53 binding alone, without ubiquitination, not only fails to inhibit p53, but actually further enhances p53 activity toward each of these targets compared to the complete absence of Mdm2. Finally, we show that binding of Mdm2^C462A^ to p53 enhances the interaction between p53 and the acetyltransferase CBP/p300, suggesting a mechanism for the enhanced p53 activity.

## Results

### Mdm2^C462A^ enhances p53 transcriptional activity

**Figure 1:**
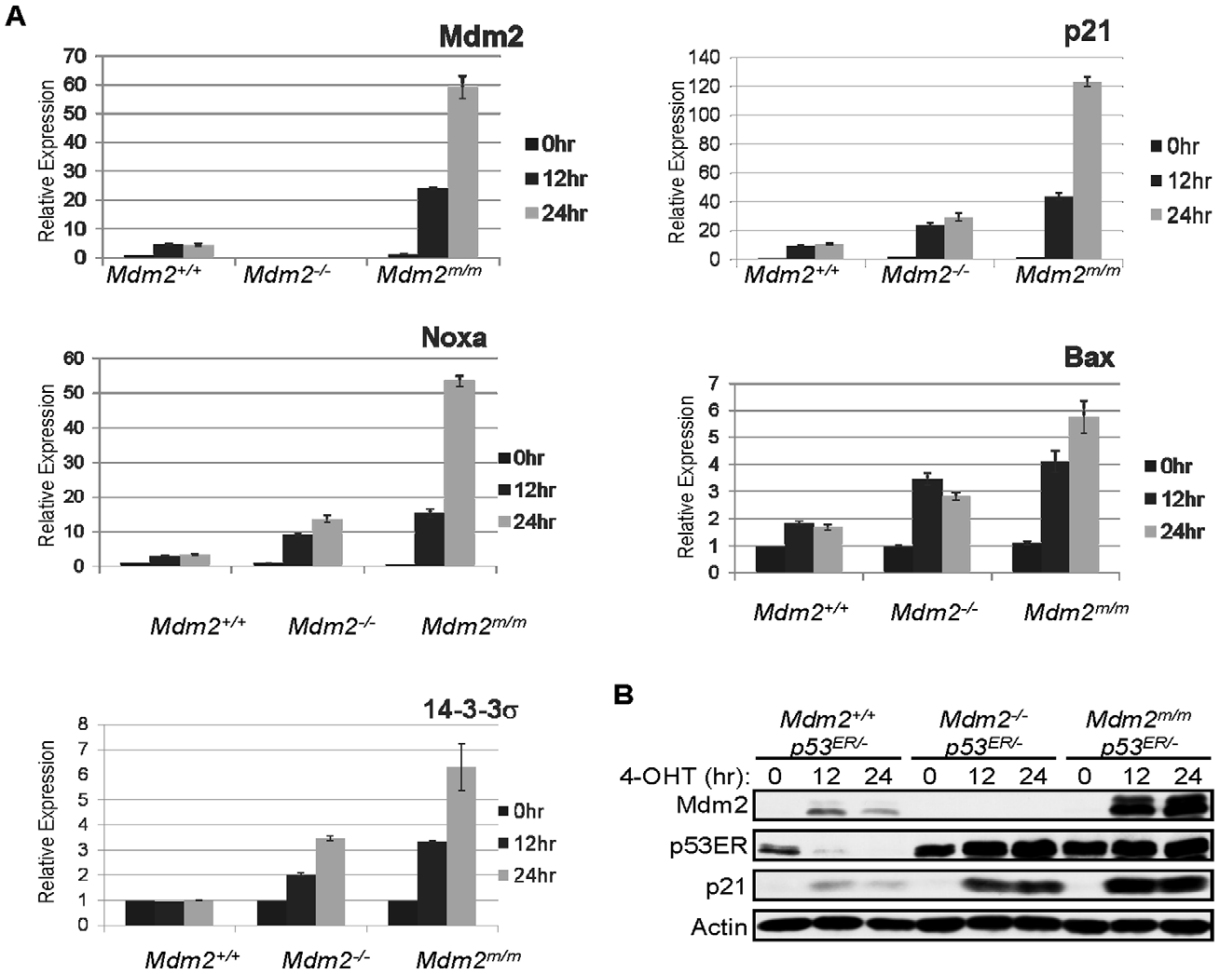
A) Quantitative real-time PCR analysis of p53 target genes in MEF cells pre-treated with 4-OHT for 24 hours to activate p53^ER^. Values represent an average of three samples measured relative to GAPDH, and error bars indicated standard deviation. All samples are of the genotype *p53^ER/−^* with Mdm2 status as indicated below graph. B) Western blot analysis of p21 expression in MEF cells of indicated genotypes at 0, 12, and 24 hours following treatment with 4-OHT to activate p53^ER^. Actin is shown as loading control.

First, we examined the effect of Mdm2^C462A^ on p53's transcriptional activity *in vivo* using MEF cells. Mice harboring the Mdm2^C462A^ mutation are not viable due to unchecked p53 activity [9]. To avoid this complication, the mice were intercrossed with mice harboring an inducible p53 (p53^ER^), in which p53 is fused with a portion of the estrogen receptor protein, rendering it inactive until treatment with the estrogen mimic, 4-hydroxytamoxifen (4-OHT) [12]. *Mdm2^m/m^*; *p53^ER/−^* mice are viable, and MEF cells from these mice can be used for studies requiring both mutant Mdm2 and active p53, as 4-OHT can be added to cultured MEF cells to induce p53 activation. To assess the effect of the C462A mutation on p53 activity, MEF cells from *Mdm2^+/+^*; *p53^ER/−^*, *Mdm2^−/−^*; *p53^ER/−^*, and *Mdm2^m/m^*;*p53^ER/−^* mice were treated with 4-OHT to reactivate p53 and were lysed after zero, 12, or 24 hours. RNA was isolated from each sample and subjected to RT-PCR to assess transcription of the p53 targets Mdm2, p21, Bax, Noxa, and 14-3-3σ. Transcription of these genes was elevated in the mutant MEFs compared to wild-type cells, confirming Itahana et al.'s finding that the RING C462A mutation renders Mdm2 unable to suppress p53 activity. Surprisingly, however, these p53 targets were expressed to a greater extent in MEFs expressing Mdm2^C462A^ than in Mdm2-null cells. Transcription was elevated in *Mdm2^m/m^* MEFs compared to *Mdm2^+/+^* and *Mdm2^−/−^* MEFs at both the 12-hour and 24-hour time points for the five p53 targets examined (Fig. 1A), indicating that the ubiquitination-deficient Mdm2^C462A^ protein not only fails to inhibit p53's transcriptional activity, but enhances it compared to lack of Mdm2.

To determine whether the increase in transcription correlated with increased expression of protein, levels of p21 were assessed by western blotting. MEF cells treated as described above were lysed with 0.5% NP-40 lysis buffer and resolved by SDS PAGE. Relative levels of p53, Mdm2, and the p53 target p21 were assessed by western blotting. The p21 protein level was elevated in *Mdm2^m/m^;p53^ER/−^* MEFs compared to *Mdm2^+/+^;p53^ER/−^* MEFs and *Mdm2^−/−^;p53^ER/−^* MEFs (Fig. 1B). It should be noted that the reduced p53 level in *Mdm2^+/+^;p53^ER/−^* MEFs is due to the Mdm2-p53 negative feedback loop; activation of p53 by administration of 4-OHT leads to enhanced transcription of Mdm2, which in turn targets p53 for degradation. This Mdm2-mediated degradation of p53 is absent in both *Mdm2*-null MEFs and those with the C462A mutation, which renders Mdm2 incapable of degrading p53.

Together, these data show that the Mdm2 C462A RING domain mutation results in increased p53 transcriptional activity, suggesting that Mdm2-p53 binding alone, without ubiquitination of p53, not only fails to suppress p53, but leads to enhanced p53 activity.

### Mdm2^C462A^ facilitates binding between p53 and CBP/p300

**Figure 2:**
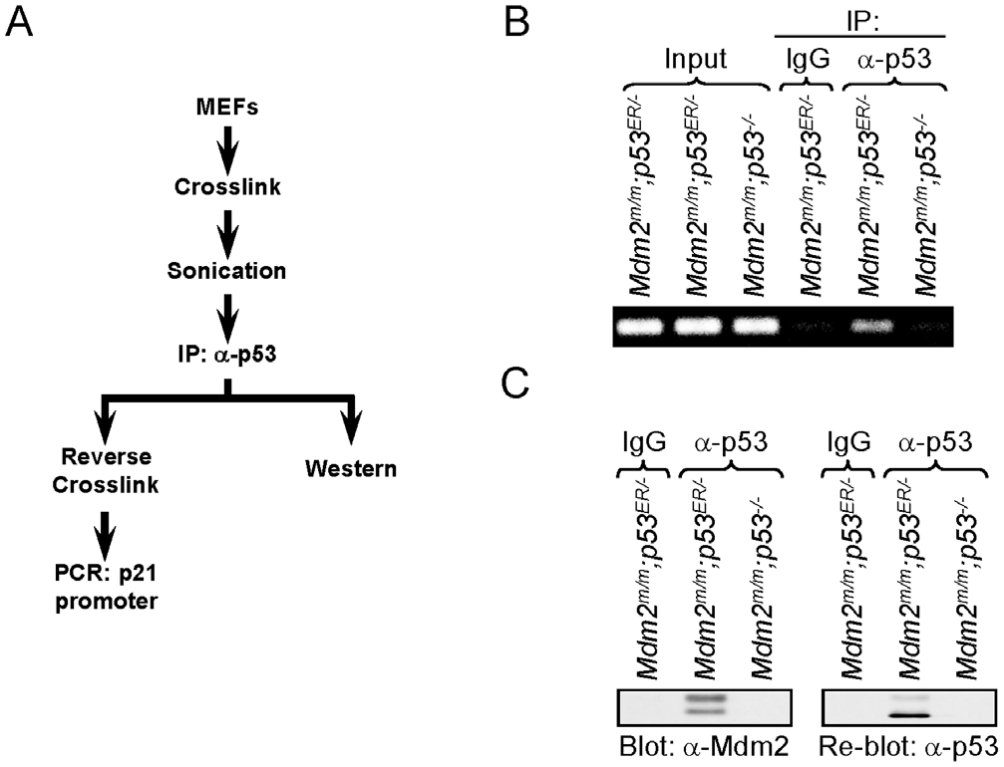
A) Schematic depicting chromatin immunoprecipitation (ChIP) analysis carried out to assess Mdm2-p53 binding on the promoter of the p53 target, p21. MEF cells were pre-treated with 4-OHT for 24 hours to induce activation of p53^ER^. Cells were crosslinked using formaldehyde, sonicated to shear chromatin, and immunoprecipitated with p53 antibody. A portion of each sample was subject to reverse crosslinking followed by PCR amplification targeting a region of the p21 promoter, while another portion was used for western blotting to assess the p53-Mdm2 interaction. B) p21/*CDKN1A* promoter was PCR amplified and resolved in 1% agarose gel following immunoprecipitation with p53 antibody and reverse crosslinking as shown in (A). C) Western blot following immunoprecipitation with p53 antibody as shown in (A). Membrane was blotted for Mdm2, stripped, and re-blotted for p53. Note that a band representing Mdm2 is present in the sample immunoprecipitated with p53 antibody but not in p53-null cells, and not following immunoprecipitation with IgG.

We explored potential mechanisms for the increased p53 activity observed in cells with the *Mdm2* C462A mutation. Mdm2 has been thought to inhibit p53's transcriptional activity by interacting with p53 on its target gene promoters and masking the transactivation domain of p53. As shown above, Mdm2^C462A^ retains its ability to interact with p53, yet does not suppress p53 activity [9]. However, it is possible that the mutant Mdm2 may not interact with p53 while located on p53's target gene promoters and is unable to control p53 activity due to this defect. To rule out this possibility, it is essential to determine whether the Mdm2^C462A^-p53 interaction can take place on the promoter of a p53 target gene. To address this directly, chromatin immunoprecipitation (ChIP) analysis was carried out to assess p53-Mdm2 binding on the p21 promoter in *Mdm2^m/m^;p53^ER/−^* MEFs cells. *Mdm2^m/m^;p53^−/−^* cells were included as a negative control. The cells were incubated with 4-OHT for 24 hours to activate p53, and formaldehyde was applied to crosslink proteins to DNA. The cells were lysed, sonicated to shear DNA, and immunoprecipitated with p53 antibody or IgG (negative control). A subset of each sample was resolved by SDS PAGE and western blotting, while another portion was subject to reverse crosslinking and PCR targeting the p21 (*CDKN1A*) promoter (Fig. 2A). PCR product indicating presence of the p21 promoter was detected equally in all three input samples, but following immunoprecipitation with p53 antibody, was present only in the sample from *Mdm2^m/m^;p53^ER/−^* MEFs. DNA from the p21 promoter was not detected in *Mdm2^m/m^;p53^ER/−^* MEFs immunoprecipitated with IgG or in *p53*-null MEFs immunoprecipitated with p53 antibody (negative controls) (Fig. 2B). Western blotting of the samples showed that both Mdm2 and p53 were present in *Mdm2^m/m^;p53^ER/−^* MEFs immunoprecipitated with p53 antibody. No Mdm2 or p53 was detected in control samples immunoprecipitated with IgG alone or from *p53*-null MEFs (Fig. 2C). These data indicate that Mdm2^C462A^ interacts with p53 on the p21 gene promoter.

**Figure 3:**
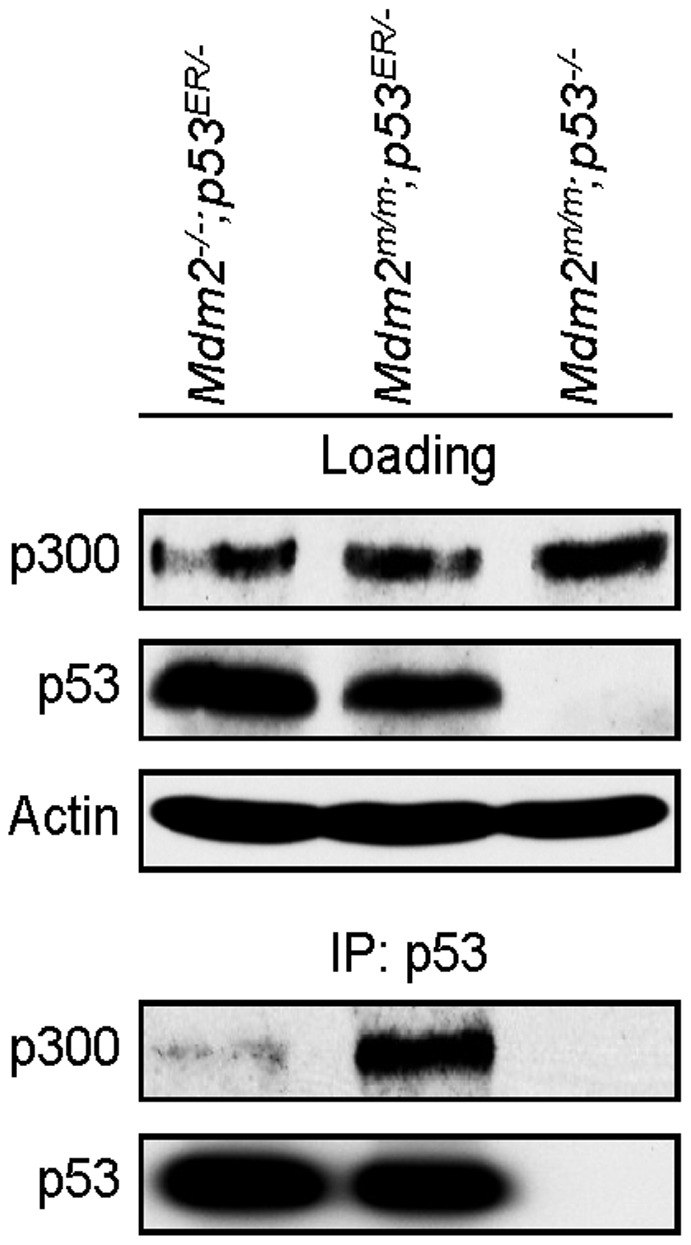
Immunoprecipitation and western blotting of MEF cell lysates 24 hours after administration of 4-OHT. Note that the p53-p300 interaction is enhanced in the *Mdm2^m/m^* MEFs compared to *Mdm2*-null MEFs despite similar immunoprecipitation of p53 and equivalent loading for p300.

We next considered potential mechanisms for the paradoxical observation that p53 activity was enhanced in the *Mdm2^m/m^* MEFs compared to *Mdm2*-null MEFs. p53 activity can be dramatically increased by acetylation, and p53 is well-known to be acetylated by its transcription cofactor, the acetyltransferase CBP/p300 [13], [14]. In response to p53-activating stressors, p300 acetylates lysine residues in p53's DNA binding domain, strongly stimulating p53's sequence-specific interaction with DNA [13], [14], [15], [16], [17], [18]. As examination of acetylation of endogenous p53 in MEF cells presents a technical challenge, we determined instead whether the C462A mutation could affect the interaction between p53 and p300. MEF cells of the genotypes *Mdm2^+/+^;p53^ER/−^*, *Mdm2^m/m^;p53^ER/−^*, and *Mdm2^m/m^;p53^−/−^* were lysed with 0.1% NP-40 lysis buffer, immunoprecipitated with p53 antibody, resolved by SDS-PAGE, and blotted for p300 and p53. The presence of the C462A mutation greatly enhanced the interaction between p53 and p300, as evidenced by a stronger band representing p300 after immunoprecipitating p53-containing complexes in the *Mdm2^m/m^;p53^ER/−^* MEFs compared to the *Mdm2^+/+^;p53^ER/−^* MEFs. No p300 was immunoprecipitated in the *p53*-null negative control sample (Fig. 3). This result indicates that Mdm2^C462A^ promotes p300-p53 binding. Because p300 is well-known to activate p53 through acetylation [13], [15], [16], [17], and increased p53-p300 interaction is associated with enhanced acetylation [19], the effect of the C462A mutation on p300-p53 binding provides an explanation for the excess p53 activity observed in *Mdm2^m/m^* MEFs compared to *Mdm2*-null MEFs.

## Discussion

This work provides further evidence that Mdm2 harboring a point mutation that abrogates its E3 ubiquitin ligase activity without affecting Mdm2-p53 binding is not capable of suppressing p53 activity. We show that Mdm2^C462A^ fails to repress transcription of five target genes, yet retains its ability to interact with p53 on the promoter of its target gene p21/*CDKN1A*. These data, complementing the 2007 study by Itahana et al. [9], challenge the long-held belief that Mdm2 can suppress p53 activity merely by binding to p53 and masking its transactivation domain. We show, along with the aforementioned study, that an intact E3 ubiquitin ligase activity is necessary for Mdm2 to inhibit p53 *in vivo* under conditions of endogenous protein expression. These findings have implications for the development of pharmaceuticals targeting Mdm2, demonstrating that it is not necessary to disrupt Mdm2-p53 binding in order to release p53 activity.

Our data show that Mdm2^C462A^ not only fails to suppress p53 activity, but, surprisingly, the mutant protein yields greater p53 activation than does the complete absence of Mdm2. This study also uncovered a potential mechanism for the increased p53 activity, showing that the C462A mutation facilitates binding between p53 and the acetyltransferase CBP/p300. The p300-mediated acetylation of p53 has been well-established to activate p53 *in vivo* [13], [15], [16], [17]. Interestingly, wild-type Mdm2 is known to inhibit p300-mediated acetylation of p53 through formation of an Mdm2-p53-p300 ternary complex [18], [20], [21], whereas our data show that a single point mutation in Mdm2's RING domain conveys an opposing effect, leading to enhancement of the p53-p300 interaction and increased p53 activity. This implies that an intact RING domain may be necessary for Mdm2's inhibition of p300-mediated acetylation of p53.

**Figure 4:**
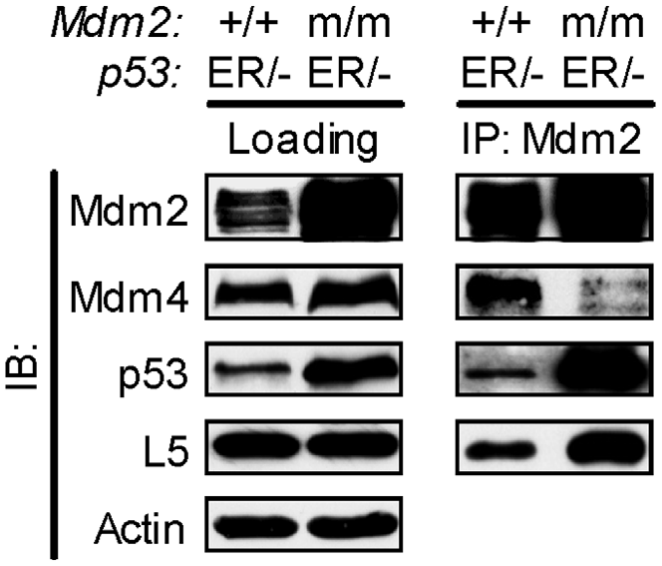
Interaction between Mdm2 and Mdmx is impaired in MEFs with Mdm2^C462A^ compared to those with wild-type Mdm2. Immunoprecipitation and western blotting were carried out 24 hours after administering 4-OHT to activate p53^ER^. Actin is shown as a loading control. Note that the interaction between Mdm2 and its known binding partner L5 is not disrupted by the C462A mutation.

**Figure 5:**
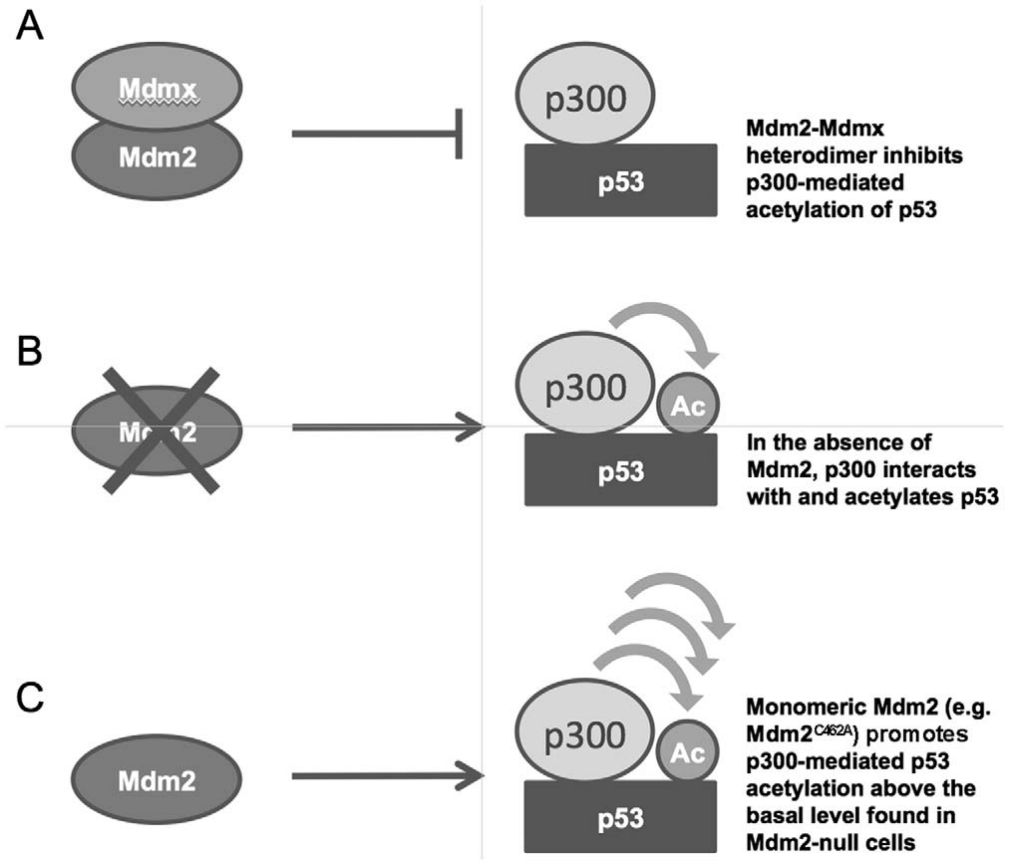
A potential mechanism for differential regulation of p53 transcriptional activity by wild-type Mdm2 and Mdm2^C462A^. A) Heterodimerization between wild-type Mdm2 and Mdmx is necessary for inhibiting the p53-p300 interaction and suppressing p300-mediated acetylation of p53, reducing p53 activity. B) Absence of Mdm2 permits p300-p53 interaction, allowing p300-mediated acetylation of p53, and thereby enhancing p53 transcriptional activity compared to that in Mdm2-positive cells. C) Mdm2^C462A^ cannot heterodimerize with Mdmx and, therefore, fails to inhibit the p53-p300 interaction, allowing enhanced p300-mediated acetylation and activation of p53. In addition, monomeric Mdm2 (such as RING mutant Mdm2^C462A^) promotes p300-p53 binding to further enhance p300-mediated p53 acetylation beyond that which occurs in Mdm2-null cells.

How might disruption of Mdm2's RING domain enhance the p53-p300 interaction? Is it due to lack of E3 ubiquitin ligase activity, or is it caused by another essential function of the RING domain? E3 ubiquitin ligase activity is not likely to be essential for inhibiting p53-p300 binding, as Mdm2's sister protein, Mdmx, lacks E3 ligase activity yet has been shown to inhibit p300-mediated acetylation of p53. It is possible that another function of the RING domain influences the interaction. One hypothesis is that formation of an Mdm2-Mdmx heterodimer may be necessary for Mdm2 to inhibit p300, as the RING domain was shown to mediate this heterodimerization [22], and Mdmx inhibits p300-mediated acetylation of p53 [23]. That is, the heterodimer may be more efficient at inhibiting p300-p53 binding than Mdm2 or Mdmx alone. To determine whether the RING point mutation affects Mdm2-Mdmx binding *in vivo*, we carried out a co-immunoprecipitation (co-IP) for Mdm2 in *Mdm2^+/+^*;*p53^ER/−^* and *Mdm2^m/m^*;*p53^ER/−^* MEF cells. We found that the C462A RING mutation disrupts the interaction between Mdm2 and Mdmx (Fig. 4), indicating that an intact RING domain is necessary for Mdm2-Mdmx heterodimerization *in vivo*. Thus, it is possible that the enhanced p53 activity and p300-p53 interaction produced by Mdm2^C462A^ may stem from its inability to heterodimerize with Mdmx. We present a hypothesized model in which the Mdm2-Mdmx heterodimer inhibits p300-p53 binding *in vivo*, while monomeric Mdm2 promotes this interaction. In this model, heterodimerization between Mdm2 and Mdmx blocks p300-p53 binding and p300-mediated acetylation of p53. In Mdm2-null cells, this inhibition is released, permitting p300 to interact with and acetylate p53. When Mdm2 exists as a monomer rather than a heterodimer, as is the case, presumably, with Mdm2^C462A^, (if not able to dimerize with Mdmx, it is also not likely to dimerize with itself), not only is it unable to inhibit p300-mediated acetylation of p53, but the monomeric Mdm2 further enhances this acetylation beyond the basal level found in Mdm2-null cells. This may be a result of monomeric Mdm2 bridging together p300 and p53, as Mdm2 is known to interact with both of these proteins [20], [21]. We speculate that the Mdm2-Mdmx heterodimer is not able to promote p300-p53 binding, perhaps due to bulkiness or another inherent difference between the monomer and the heterodimer (Fig. 5). It should be noted that this study does not specifically differentiate between Mdm2-Mdmx heterodimerization and Mdm2-Mdm2 homodimerization. Ultimately, further research will be needed to determine whether the effect of Mdm2^C462A^ on the p300-p53 interaction is mediated by its ability to heterodimerize with Mdmx, or by another mechanism.

Nonetheless, these data show that Mdm2-p53 binding alone is not sufficient for inhibiting p53 activity or p53's interaction with the acetyltransferase p300, enhancing our understanding of the complex regulation of the p53 tumor suppressor pathway.

## Materials and Methods

### Mouse Generation and Maintenance

Mice were generated, maintained, and genotyped as described previously [9].

### Quantitative Real-Time PCR

RNA was extracted using the RNeasy Mini Kit (Qiagen) according to the manufacturer's instructions. cDNA was synthesized using SuperScript III (Invitrogen). Quantitative real-time PCR was performed with SYBR Green using the Applied Biosystems 7900HT Fast Real-Time PCR System, and data was collected and exported with SDS 2.2.2. Relative expression was calculated using GAPDH as an internal control.

### Cell Culture

MEF cells were cultured in a 37°C incubator with 5% CO2 in DMEM supplied with 10% fetal bovine serum (Gibco) and penicillin (100 IU/ml)/streptomycin (100 µg/ml). To activate p53^ER^, 100 nM 4-hydroxytamoxifen (4-OHT; Sigma) dissolved in ethanol was added to the culture medium.

### Protein Analysis

Cells were lysed in 0.1% NP-40 buffer for immunoprecipitation and 0.5% NP-40 buffer for straight western blotting. Procedures and conditions for immunoprecipitation and immunoblotting were described previously [24]. The following antibodies were purchased commercially: mouse monoclonal Mdm2 (2A-10 and 4B11, Calbiochem), p53 (NCL-505, Novocastra; DO-1, Lab Vision/Neomarkers), actin (MAB1501, Chemicon International), goat polyclonal p53 (FL-393; Santa Cruz), and rabbit polyclonal p53 (CM5, Novocastra). Rabbit polyclonal p21 antibody was a gift from Dr. Yue Xiong (UNC-Chapel Hill). Rabbit polyclonal antibodies to L5 and L11 were described previously [25].

### Chromatin Immunoprecipitation (ChIP) Analysis

MEF cells were crosslinked using 1% formaldehyde for 10 min at 37°C and washed with PBS. Crosslinking was stopped with 0.125 M glycine in PBS, cells were washed in PBS, centrifuged for 5 min at 1200 rpm, and pellets were resuspended in Lysis Buffer A (10 mM Hepes pH 7.5, 0.5% NP40, 1.5 mM MgCl_2_, 10 mM KCl, 0.5 mM DTT). Tubes were rotated at 4°C for 30 min and spun down at 13,000 rpm for 5 min. Proteins not crosslinked to chromatin were removed, and pellets were resuspended in Lysis Buffer B (20 mM Hepes pH 7.5, 25% Glycerol, 0.5% NP40, 0.5% Triton X-100, 0.42 M NaCl, 1.5 mM MgCl_2_, 0.2 mM EDTA, 1 mM PMSF, protease inhibitor, and Na_3_VO_4_). Lysates were sonicated for 10 seconds at 1 min intervals a total of 4 times using a Branson Digital SONIFIER® (Model 250, 450) and Branson Sound Enclosure (Model SSE-1). Samples were centrifuged for 10 min at 13,000 rpm at 4°C. The supernatant was collected, the protein concentration was measured, and the supernatant was mixed with an equal volume of dilution buffer (0.01% SDS, 1.1% Triton X-100, 1.2 mM EDTA, 16.7 mM Tris pH 8.0, 167 mM NaCl, 1 mM PMSF, protease inhibitor, and Na_3_VO_4_). Samples were precleared with Protein G Agarose with Salmon Sperm DNA (Upstate/Millipore, Cat. # 16-201) for 30–60 min at 4°C with agitation. Samples were spun down at 3000 rpm for 2–5 min at 4°C, and supernatant was collected, with 5% reserved for use as a loading control for western blotting. Samples were incubated with antibodies overnight (goat anti-p53 FL393 or goat IgG) using 0.6 µg of antibody per 1 mL sample. 20 µL of Protein G Agarose/Salmon Sperm DNA was added and samples were incubated for 1 hr, spun at 3000 rpm at 4°C for 3 min, and washed sequentially with the following buffers at 4°C: TSE I (0.1% SDS, 1% Triton X-100, 2 mM EDTA, 150 mM NaCl, 20 mM Tris-HCl pH 8.0), TSE II (0.1% SDS, 1% Triton X-100, 2 mM EDTA, 500 mM NaCl, 20 mM Tris-HCl pH 8.0), TSE III (0.25 M LiCl, 1% NP40, 1% deoxycholate, 1 mM EDTA, 10 mM Tris-HCl pH 8.0), and twice with TE (10 mM Tris-HCl pH 8.0, 1 mM EDTA). A portion of beads was resuspended in 1× sample buffer for western blotting. For anticrosslinking and PCR, samples were eluted 3× with 75 µL of elution buffer (1%SDS, 0.1M NaHCO3, 1 mM DTT), vortexed briefly, and incubated at room temperature for 15 min with rotation. Eluates were pooled (200 µL) and 8 µL of 5 M NaCl added. Anticrosslinking was performed at 65°C for 6 h to overnight. Samples were treated with 4 µL of 0.5 M EDTA, 4 µL of 2 M Tris-HCl pH 6.8, 2 µL of 10 mg/ml Proteinase K, 2 µL of 10 mg/ml RNAse A, and incubated for 1 h at 45 C. DNA was recovered with a QiaQuick PCR Purification Kit (Qiagen) and eluted with 50 µL of 10 mM Tris-HCl pH 8.5. PCR was carried out using the following primers for p21 promoter: Promoter mp21 F1 (Forward; CCAGAGGATACCTTGCAAGGC) and Promoter mp21 R1 (Reverse; TCTCTGTCTCCATTCATGCTCCTCC) [26]. Samples were resolved on 1% agarose gel.
